# Predicting Performance and Plasticity in the Development of Respiratory Structures and Metabolic Systems

**DOI:** 10.1093/icb/icu018

**Published:** 2014-05-07

**Authors:** Kendra J. Greenlee, Kristi L. Montooth, Bryan R. Helm

**Affiliations:** *Department of Biological Sciences, North Dakota State University, Fargo, ND 58102, USA; ^†^Department of Biology, Indiana University, Bloomington, IN 47405, USA

## Abstract

The scaling laws governing metabolism suggest that we can predict metabolic rates across taxonomic scales that span large differences in mass. Yet, scaling relationships can vary with development, body region, and environment. Within species, there is variation in metabolic rate that is independent of mass and which may be explained by genetic variation, the environment or their interaction (i.e., metabolic plasticity). Additionally, some structures, such as the insect tracheal respiratory system, change throughout development and in response to the environment to match the changing functional requirements of the organism. We discuss how study of the development of respiratory function meets multiple challenges set forth by the NSF Grand Challenges Workshop. Development of the structure and function of respiratory and metabolic systems (1) is inherently stable and yet can respond dynamically to change, (2) is plastic and exhibits sensitivity to environments, and (3) can be examined across multiple scales in time and space. Predicting respiratory performance and plasticity requires quantitative models that integrate information across scales of function from the expression of metabolic genes and mitochondrial biogenesis to the building of respiratory structures. We present insect models where data are available on the development of the tracheal respiratory system and of metabolic physiology and suggest what is needed to develop predictive models. Incorporating quantitative genetic data will enable mapping of genetic and genetic-by-environment variation onto phenotypes, which is necessary to understand the evolution of respiratory and metabolic systems and their ability to enable respiratory homeostasis as organisms walk the tightrope between stability and change.

## Background and motivation

The scaling laws that relate metabolic rates to mass suggest that metabolic performance is well predicted by a power law that allometrically scales metabolic traits with mass to the power of 3/4 or 2/3, indicating that large organisms respire less per unit mass than do small organisms (Kleiber 1947; Hemmingsen 1960; Schmidt-Nielsen 1984; Brown and West 2000; Savage et al. 2004; Chown et al. 2007; Sibly et al. 2012). Although this scaling law can be remarkably predictive across many orders of magnitude of mass, there is substantial evidence that metabolic rate is a dynamic trait and that its relationship to mass can vary depending on taxonomic group, organismal mode of temperature regulation, activity level, and the environment, as well as within an individual throughout ontogeny [reviewed by Glazier (2005)]. Among closely related species and within species, there is ample variation in metabolic rate above and beyond the variance explained by mass. Thus, not only can the exponent describing how metabolism scales with mass (*b*)—the slope of the relationship between log(metabolic trait) and log(mass)—vary, but the mass-specific metabolic rate can also vary among individuals as a function of genotype, life stage, and the environment. To explain variation and predict plasticity in metabolic rate, we need quantitative models that explicitly incorporate those variables that underlie metabolic rate beyond mass, including genetic and environmental effects on the development of systems underlying energetic supply and demand.

Maintaining energy homeostasis across development is a fundamental aspect of organismal form and function. As the organism develops, it grows larger and experiences changing functional and environmental challenges that influence the relative demand for oxygen (O_2_) and metabolic substrates. As a consequence, respiratory structures and metabolic systems are likely to change throughout development and in response to the environment. The study of the development of metabolic performance in the context of the environment thus provides an opportunity to dissect mechanisms and develop models that explain how homeostatic physiological systems are inherently dynamic, both to ensure stability at the level of energy homeostasis and to allow change in metabolic set-points and respiratory structures in response to the environment. The latter process can occur both on shorter timescales during which the environment elicits rapid and reversible strategies of acclimation and on longer timescales during which the environmental context of development may lead to alternative physiological strategies in later life stages. Although many factors, including composition of membranes and proton leaks in plasma membranes, determine the steady-state metabolism of an organism, particularly in endotherms (Hochachka and Somero 2002; Hulbert and Else 2005), here we focus on the ontogeny of metabolic rate in developing insects as a function of the energy (i.e., ATP) demands of the cell. We also detail the respiratory structures (the tracheae) and metabolic systems that supply the O_2_ and metabolic substrates to meet these demands. Insect development is a good model for understanding the dynamics of energy demand and supply, because larval growth is intensive (Church and Robertson 1966; Goodman et al. 1985; Greenlee and Harrison 2004a; Blossman-Myer and Burggren 2010; Sears et al. 2012) and must be balanced by the storage of energy, which is required to successfully complete metamorphosis in holometabolous insects.

Much progress has been made in using quantitative models to describe biomechanical phenomena, locomotor processes, and neural-response systems, and applications of control theory that take a top–down approach have been particularly useful (Cowan et al. 2014; Roth et al. 2014). We suggest that application of control theory to the development of respiratory structures and metabolic systems will provide insight into the factors, beyond mass, that determine metabolic performance and plasticity. There is an extensive literature and numerous reviews on metabolic scaling (see above for references); here we highlight and discuss recent data on the development of the tracheal and metabolic systems in the larval forms of several species of insects that could contribute to building predictive models of metabolic performance. We focus primarily on the fruit fly, *Drosophila melanogaster*, the grasshopper, *Schistocerca americana*, and the tobacco hornworm, *Manduca sexta*, because these species have the most complete datasets. When applicable, we note important findings in other species and suggest other taxonomic groups within the insects that are important for further development. We also highlight the importance of incorporating genetic variation into experiments and models to better understand the evolution of metabolic performance and plasticity. The ability to predict metabolic performance, its response to the environment, and its capacity to evolve is of key importance for understanding how changing climate will influence the roles of insects in ecosystem functions and their roles as pests and pollinators (Williams et al. 2010). Furthermore, applying quantitative approaches within a comparative framework may facilitate the development of theory to explain general principles of organismal structure and function.

## Development of the insect tracheal respiratory system

### Structure and function of the tracheal respiratory system

**Fig. 1:**
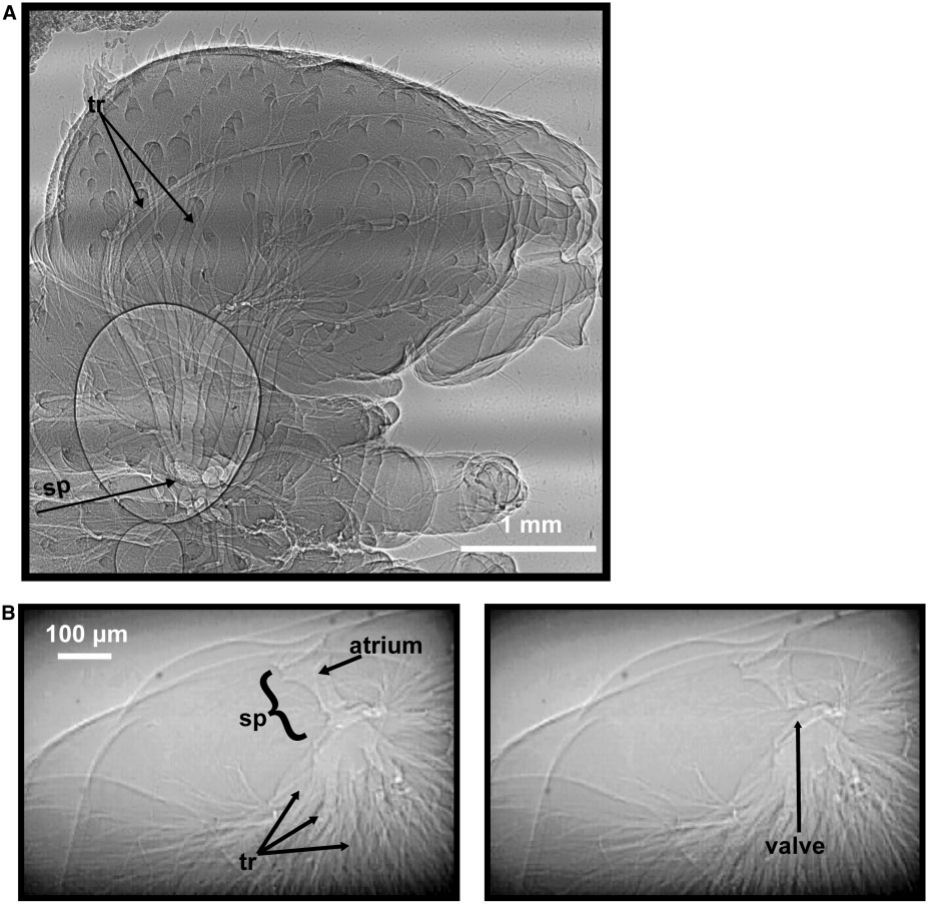
Synchrotron x-ray images of *M. sexta*, highlighting structures of the tracheal system. (**A**) Lateral view of a first instar hatchling’s head showing one spiracle (sp) covered by the sieve plate and numerous branching tracheae (tr). (**B**) Cross-sectional views of a fifth instar caterpillar, showing the spiracle, the atrium, and the muscular spiracular valve. The left panel shows the valve in the open state, and the right panel shows the valve in the closed state.

The respiratory system of insects comprises three major parts (reviewed in Chapman 1998; and Harrison 2009): (1) spiracles, valved structures that connect the respiratory system to the atmosphere, occur in pairs on body segments and can vary in number across species and life stages (Fig. 1). Opening and closing of spiracles (Fig. 1B) are controlled by muscles that are driven by impulses from the ventral nerve cord. (2) Tracheae, a network of tubes, branch from the spiracles into increasingly smaller tubes (Fig. 1). Some insect species and life stages have enlarged, flexible tracheae called air sacs that act as a bellows, driving airflow through the tracheal system. Tracheae eventually reach the tissues as tracheoles, where they transport O_2_ and CO_2_ in the gaseous phase. The distinction between small tracheae and tracheoles is sometimes unclear. (3) Tracheoles are air-filled channels made from single tracheolar cells (Harrison 2009). They are blind-ended, fluid-filled, and usually between 0.1 and 1 µm in diameter (Harrison 2009). O_2_ diffuses across the tracheolar fluid and cell membranes to be used for cellular respiration. Levels of tracheolar fluid have been shown to decrease during hypoxia, presumably decreasing the distance across which O_2_ must diffuse to reach the tissues (Wigglesworth 1931).

Development of the insect tracheal respiratory system begins during embryogenesis from clusters of ectodermal cells located on each side of the thoracic and abdominal body segments (Manning and Krasnow 1993). These clusters of cells invaginate and elongate, directed by expression of the genes “branchless” and “breathless” that encode the fibroblast growth factor (FGF) and FGF-receptor homologs, respectively (Samakovlis et al. 1996; Sutherland et al. 1996). Thus, the tracheal system is contiguous with the exoskeleton, and major parts of it are shed as the insect molts to its next developmental stage or instar. This has long been considered a constraint on within-instar development, as the insect increases in body size and therefore in demand for O_2_, whereas the O_2_ supply system is fixed. However, recent data reveal that tracheal mass and volume increase during the penultimate instar in *M. sexta* (Helm and Davidowitz 2013). Other studies show that tracheae appears to stretch as insects grow, indicating a possible safety margin for tracheal supply (Callier and Nijhout 2011). Nevertheless, insects remain O_2_-limited as they near the end of an instar (Greenlee and Harrison 2004b, 2005; Callier and Nijhout 2011), suggesting that any increases in the size of the tracheal system may not be enough to overcome the intensive energy demands of growth.

Transport of gases in the gaseous phase is advantageous. The respiratory system is mostly air-filled and, as such, it is lightweight and allows high rates of gas exchange. Flying insects have the highest rates of gas exchange recorded for locomoting animals (Harrison and Roberts 2000). Insects are incredibly tolerant of hypoxia and anoxia, traits that are also attributed to the efficient tracheal respiratory system. Insects are able to breathe and maintain gas exchange in atmospheres with as little as 0.5% O_2_, levels that would be lethal to mammals (Hoback and Stanley 2001; Harrison et al. 2006). Although the advantages of an air-filled tracheal system are clear, delivery of O_2_ in the gaseous phase is not without consequence, as direct exposure to O_2_ increases the likelihood of oxidative damage. However, many insects have adaptations that reduce oxidative damage to tissue, including discontinuous gas exchange (Hetz and Bradley 2005; Bradley 2006).

Gas exchange in insects occurs via both diffusion and convection. While diffusion may be adequate to sustain metabolism for very small insects (Krogh 1919), many insects, even small ones, use convection to supplement transport of gases through the tracheae (reviewed in Greenlee and Harrison 2004a). Insects generate convective flow through several mechanisms, the most common of which is abdominal pumping, during which muscular contractions of the abdomen decrease the volume of the abdomen and increase internal pressure, driving air through the system (Harrison et al. 2013). Coordination of abdominal contractions with spiracular opening and closing results in tracheal compression and generates directional airflow (Socha et al. 2008, 2010; Greenlee et al. 2009, 2013). Insects also have been documented to use auto-ventilation of the thorax and head (Miller 1960) and passive-suction ventilation (Kestler 1985). Although respiratory system functions, such as breathing frequency and tidal volume, have been shown to vary across development in insects (Miller and Mills 1976; Greenlee and Harrison 2004a, 2004b, 2005; Kirkton et al. 2012; Snelling et al. 2012), we limit our discussion to ontogenetic changes in the structures of the tracheal system.

### Ontogeny of the tracheal system

The structures of the tracheal system and their sizes can vary with life stage. For example, air sacs typically appear only in older stages, with *M. sexta* and *D. melanogaster* having air sacs only as adults (Eaton 1988; Brody 1999). Air sacs likely facilitate flight by acting as a bellows, increasing convective gas exchange (Harrison and Roberts 2000) and are hypothesized to play a role in hemolymph circulation (Wasserthal 1996; Wasserthal 2012). In growing grasshoppers, volumes of air sacs scale hypermetrically with mass (Greenlee et al. 2009), and adult grasshoppers invest significantly greater resources into the tracheal system than do younger hoppers. In addition, size of the tracheal structure (e.g., volume, mass, and diameter) may not change as predicted by body mass (Table 1). Investigation of larval, wandering *D. melanogaster* showed that tracheal diameter did not scale with body mass (Henry and Harrison 2004), although the range of masses may have been too small to infer scaling relationships. In developing grasshoppers, tracheal scaling of two dorsal transverse tracheae exhibited isometric growth in diameter, but hypermetric growth in length (Harrison et al. 2005). Across developmental stages, or instars, increases in tracheal volume are likely due to the presence of air sacs in animals older than the second instar (Greenlee et al. 2009), although other research shows a proportional increase with age in the tracheal volumes of grasshoppers’ legs, suggesting that there is an increased investment in tracheae and especially tracheoles in the femur (Hartung et al. 2004). Not much is known about the scaling of tracheoles because of their small size and the difficulty of conducting histological studies across the bodies of large insects.

**Table 1:** Mass-scaling exponents for tracheal system parameters measured throughout ontogeny.

Parameter	Scaling exponent	Species	Developmental stages covered	References
air sac volume	1.38	*Schistocerca americana*	First instar to adult	(Greenlee et al. 2009)
tracheal system volume	1.3	*Schistocerca americana*	First instar to adult	(Lease et al. 2006)
thoracic tracheal diameters	0.22, 0.27, 0.25, 0.32	*Manduca sexta*	larval instars 2–5	(Greenlee et al. 2013)
tracheal system volume	1.04	*Manduca sexta*	larval instars 3–5	(Callier and Nijhout 2011)
	0.94	*Manduca sexta*	larval instar 5	(Helm and Davidowitz 2013)
tracheal diameters	0	*Drosophila melanogaster*	larval instars	(Henry and Harrison 2004)

Scaling patterns across instars may vary from scaling patterns within an instar, due to the discontinuous growth of the tracheal system. Tracheal volume in grasshoppers increases overall throughout the lifespan of the insect (Lease et al. 2006). However, within an instar, tracheal volumes decrease (Clarke 1957; Lease et al. 2006). The decreases within an instar are likely due to increased tissue mass with limited growth of sclerotized, exoskeletal structures, which causes compression of femoral air sacs in grasshoppers (Kirkton et al. 2012). In *M. sexta*, across three larval instars, tracheal volumes scale isometrically with mass, yet within each instar tracheal volumes decrease slightly (Callier and Nijhout 2011).

**Fig. 2:**
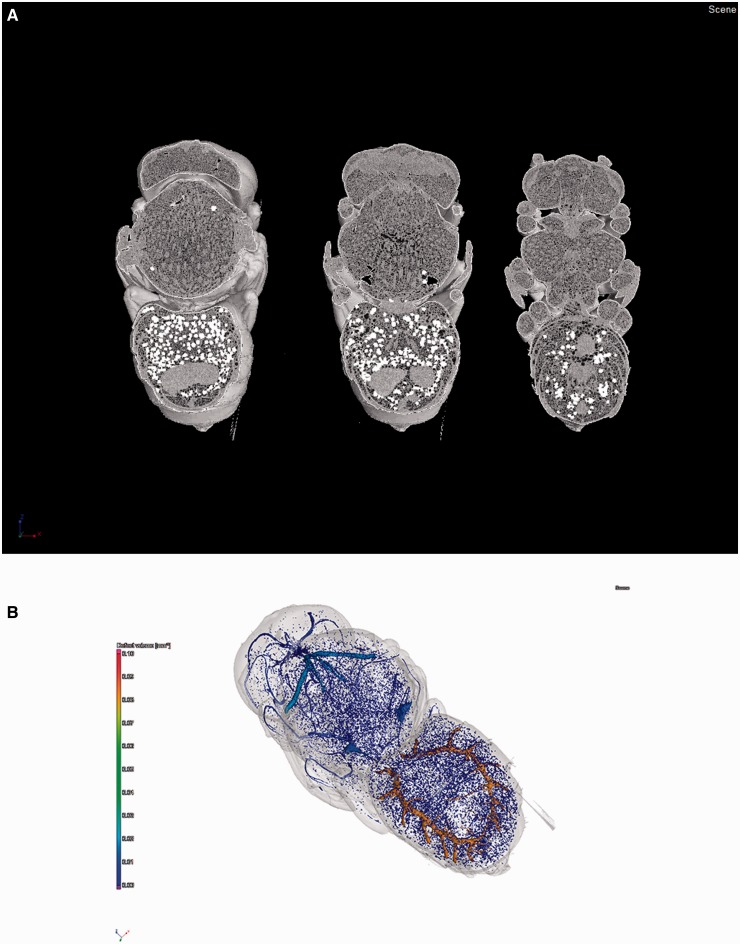
Micro-computed tomography images of a pupal alfalfa leafcutting bee, *Megachile rotundata*, 14 days after metamorphosis was initiated. (**A**) Digital cross-sections of the bee after imaging and reconstruction. (**B**) Artificially colored image showing air-filled tracheae that were identified after reconstruction. Color scale indicates volume of identified tubes.

To develop accurate models that generate meaningful predictions, we need a complete dataset of tracheal volume and its growth pattern across an entire lifespan in at least one species. Many studies of tracheal volume have employed different methods that may not be very precise, such as displacement of water (Callier and Nijhout 2011), estimates of tracheal mass (Helm and Davidowitz 2013), or washout of inert gas (Lease et al. 2006). Recently, the method of microcomputed tomography (microCT) has been used to measure volumes of tracheal systems (Socha and De Carlo 2008; Shaha et al. 2013), providing a non-destructive and repeatable method for accurate measurement of tracheal volumes from numerous samples (Fig. 2). While microCT can detect tubes as small as 1 µm in diameter, depending on the resolution of the CT system, tracheal volume may still be underestimated. However, applying this method to obtain tracheal volumes as a function of development for a few key species and matching those with metabolic measures (see below) nonetheless provides an opportunity for us to better understand and predict changes in metabolic performance during development and in response to the environment.

### Plasticity of the tracheal system in response to the environment

Plasticity of the tracheal system during development is not well documented, as even measurements of normal development are difficult to obtain. The most common experimental manipulation is alteration of atmospheric PO_2_, coupled with measurements of tracheal diameters, lengths, and/or branching. Measurements of the volumes of tracheal systems of whole animals in response to rearing in hypoxia or hyperoxia are underway, but as of yet, unpublished. As the tracheal system is the only supply of O_2_, rearing insects in hypoxia is predicted to have significant effects on tracheal dimensions. Wigglesworth (1954) conducted one of the first studies to identify effects of O_2_ on tracheal system development, showing that kissing bugs, *Rhodnius prolixus*, reared in hypoxia developed more large tracheae than did those reared in normoxia. Similar responses have been documented in the mealworm, *Tenebrio molitor* (Locke 1958). Loudon (1989) also found that mealworms reared in hypoxia (10.5% O_2_) had cross-sectional areas of tracheal branching from spiracles that were twice as large as those of larvae reared in normoxia.

However, when we look across insect species and developmental stages, the effect of atmospheric PO_2_ on tracheal development is not as clear. Larval *D. melanogaster* reared in hypoxia exhibit no change in larval tracheal diameters (Henry and Harrison 2004), but the number of terminal tracheolar branches increases in larvae (Jarecki et al. 1999), and adults’ tracheal diameters are larger when reared in hypoxia (Henry and Harrison 2004), suggesting that the atmospheric O_2_ levels experienced by larvae impact tracheal development during metamorphosis. In contrast, *S. americana* experience no change in tracheal diameter when reared in hypoxia (Harrison et al. 2006). Measures of the tracheal parameters of *M. sexta* reared in hypoxia are lacking, as are measures of the structures/volumes of the tracheal system in whole animals.

Atmospheric O_2_ is an obvious mediator of tracheal system development, but quality of diet also affects tracheal system development. *M. sexta* reared on a low quality diet (a 60% reduction of nutrients and calories) had an increase in dry mass of the tracheal system during the fifth and final instar (Helm and Davidowitz 2013). The scaling exponent for tracheal mass also increased from 0.89 on the high quality diet to 1.16 on the low quality diet, indicating that larger animals were increasing the amount of tracheae available for oxygen delivery when reared under low-nutrient conditions (Helm and Davidowitz 2013). This result is seemingly in contrast to the obvious prediction that less nutrients would lead to a lower metabolic rate and less available ATP. However, when we examine the pathways that mediate tracheal system proliferation, we find that decreases in available ATP activate the same signaling pathways as hypoxia (Harrison and Haddad 2011). Thus, the authors hypothesized that under the low-nutrient conditions, *M. sexta* had less ATP available and tracheae increased as a compensatory response.

### Genetic effects on tracheal development

No studies have investigated the contribution of genetic variation to structures of the tracheal system throughout larval development, thus far. However, natural genetic variation is associated with variation in the size of the tracheal system in adults of the fritillary butterfly, *Melitaea cinxia* (Marden et al. 2013). Fritillary butterflies have a polymorphism in the *succinate dehydrogenase d* gene, encoding the enzyme that converts succinate to fumarate in the tricarboxylic acid (TCA) cycle. Low succinate dehydrogenase (SDH) activity activates the hypoxia signaling pathway by stabilizing the transcription factor, hypoxia inducible factor-1 (HIF-1) complex (Gimenez-Roqueplo et al. 2001). Tracheal cells are highly responsive to activation of HIF and are stimulated to branch when HIF subunits are overexpressed (Centanin et al. 2008, 2010), simulating a hypoxic environment. In the fritillary butterfly, individuals having an allelic variant with low SDH activity, have double the tracheal area in the flight muscle compared with those with other alleles (Marden et al. 2013). This is in congruence with another study showing that *D. melanogaster* with a defect in the SDHB subunit are overly sensitive to hyperoxia (Walker et al. 2006). One would predict that this mutation results in a larger tracheal system compared with that in wild-type flies, thereby providing an excess of O_2_ to tissues that may cause oxidative damage.

## Development of the underlying metabolic systems

### Metabolic systems and their function

Energy metabolism embodies the idea of walking the tightrope between stability and change. Balancing supply and demand of ATP in cells is inherently homeostatic, and yet, the changing energetic requirements across tissues, life stages, activities, and environments require that the underlying pathways of energy metabolism, and hence cellular metabolic rates, are dynamically regulated to maintain this homeostasis. Hochachka and Somero (2002) described metabolic control models in the context of the large dynamic range of the metabolic rates of muscles as the “problem (and paradox)” of how “muscles sustain both metabolic homeostasis and metabolic regulation”. The regulation of this homeostasis has largely been modeled using feedback-control circuitry in which ATP-demand pathways increase the concentration of ADP, which is then the substrate of ATP-supply pathways (Hochachka and Somero 2002; Clarke and Fraser 2004). In this way, energy demand can affect both O_2_ and carbon fluxes for ATP synthesis. Fully understanding the regulation of the supply and demand of ATP will require interfacing measurements of the tracheal delivery of O_2_ with those of the underlying metabolic systems, including the functional capacity of the mitochondria.

**Fig. 3:**
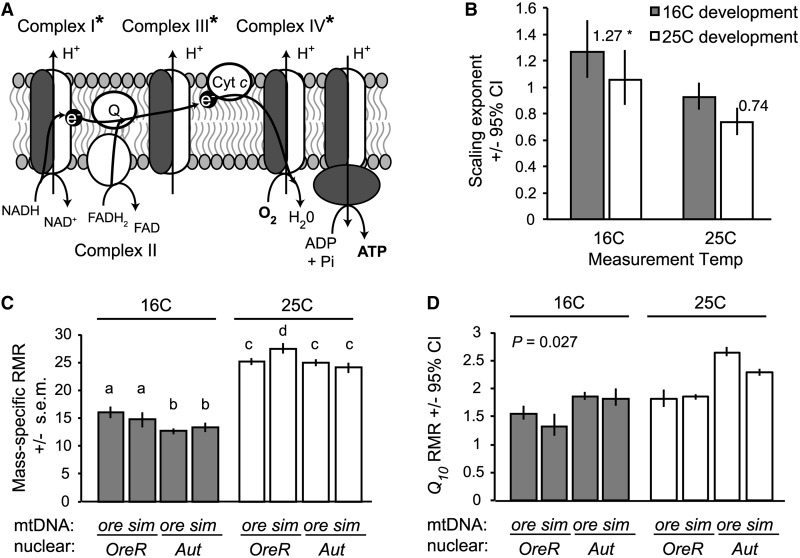
Genetic and environmental effects on metabolic scaling, mass-specific RMR, and metabolic plasticity in *D. melanogaster* larvae. (**A**) Production of ATP via OXPHOS requires interactions between gene products encoded in the nuclear genome and in the mtDNA. A specific mitochondrial-nuclear genotype disrupts protein synthesis in the mitochondria and decreases activity for the three complexes in the electron transport chain that require proteins from both genomes (indicated by an asterisk; ATP synthase activity was not measured in this study) (Meiklejohn et al. 2013). (**B**) Larvae of different mitochondrial-nuclear genotypes had similar metabolic scaling exponents, but the scaling of RMR with mass was affected both by the temperature experienced during development and by the temperature at which the larvae were measured. Flies developed and measured at 16°C had a scaling exponent that was significantly >1 (*P* < 0.05), whereas flies developed and measured at 25°C had a scaling exponent that was not significantly different from 0.75 (*P* > 0.05). (**C**) When developed and measured at 16°C, larvae with different nuclear genomes had significantly different scaling coefficients, indicating different mass-specific RMR. However, when developed and measured at 25°C, the mitochondrial-nuclear genotype that compromises OXPHOS capacity shows a significantly elevated mass-specific RMR. Letters within temperatures indicate significant differences between genotypes (*P* < 0.05, type II regression analysis). (**D**) Mitochondrial-nuclear genotype also affects the *Q_10_* of RMR (i.e., metabolic plasticity), and this effect depends upon the temperature during development, as indicated by a significant interaction between mtDNA, nuclear genome, temperature during development, and temperature during measurement (*P* = 0.027). Data are RMRs from 20 replicate pools of 5 third-instar, pre-wandering larvae per genotype and temperature treatment, as described in Hoekstra et al. (2013).

The ATP required to fuel cellular processes is supplied by aerobic and anaerobic pathways that use different substrates. In largely aerobic organisms, such as many insects, production of ATP occurs via the proton gradient established by the oxidative phosphorylation (OXPHOS) complexes in the mitochondria (Fig. 3A). Thus, the volume of O_2_ consumed (VO_2_) and CO_2_ produced (VCO_2_) are common measures of the metabolic rate associated with production of ATP in insects. However, even in largely aerobic insects, such as *D. melanogaster*, production of ATP via anaerobic pathways (e.g., those producing lactate) is a critical component of development (Tennessen et al. 2011). Different utilization of substrates during production of ATP (e.g., carbohydrate versus lipid) alters the ratio of VCO_2_ to VO_2_, the respiratory quotient (RQ). RQ can be a good indicator of variation in energy metabolism between individuals and species, but is also likely to vary within an individual as a function of developmental stage, tissue, activity level, or environment. Thus, to generate ATP supplies that balance cellular demand, metabolic systems require integrated and dynamic regulation of the protein products of two genomes, nuclear and mitochondrial (Fig. 3A), which comprise the metabolic pathways housed in both the cytoplasm and the mitochondria. Below, we discuss new data measuring these processes as a function of development, genotype, and the environment. We then highlight the need for more complete datasets from a few species to develop predictive models of metabolic rate variation and plasticity in response to the environment.

### Ontogeny and scaling of metabolic rate and demand for energy

Across organisms that vary in mass by ∼20 log cycles, a single scaling exponent (*b*) of 0.75 is a remarkably good predictor of metabolic rate (Kleiber 1947; Hemmingsen 1960; Schmidt-Nielsen 1984; Brown and West 2000; Savage et al. 2004; Chown et al. 2007). However, if the aim is to explain variation in metabolic rate among species of similar masses, between individuals in different environmental contexts, or within an individual across its lifespan, it is important to acknowledge that a scaling exponent of *b* = 0.75 is by no means the rule. Glazier (2005) extensively reviewed the literature documenting variation in *b* among taxonomic groups, life styles, activity levels, environmental contexts, and within individuals across their lifespans. In addition, the scaling coefficient that describes the mass-specific metabolic rate can vary among insect species (Chown et al. 2007), among individuals of different genotypes within a species (Montooth et al. 2003; Hoekstra et al. 2013), and across development (Greenlee and Harrison 2005; Callier and Nijhout 2011; Callier and Nijhout 2012; Sears et al. 2012). If the mass-scaling of metabolic rate is a composite function of how different components of energy metabolism, including ATP demand, scale with mass (Hochachka and Somero 2002), then we should expect that *b* will vary among species and individuals that experience different genetic, physiological, developmental, and environmental conditions.

Here, we focus on recent datasets that carefully dissect the relationship between metabolic rate and mass within and among the larval instars of insects. These datasets serve as a starting point for quantitative modeling of the development of metabolic rate that can be interfaced with modeling of the development of the tracheal system. Metabolic rates of larvae are often measured while larvae are growing, feeding, and locomoting to some extent, such that these measures may more accurately reflect routine metabolic rates that are higher than resting metabolic rates (Clarke and Fraser 2004). Comparisons between resting, routine, and maximal metabolic rates could provide insight on how basal metabolic rates and the scope for metabolic plasticity evolve (Artacho and Nespolo 2009) and whether the resting metabolic rates of organisms are over-designed to facilitate the high levels of metabolism required for active states. However, this data is currently lacking for the larval systems described below.

Several recent studies have documented that the relationship between mass and routine metabolic rate (RMR) changes within and across larval instars of the tobacco hornworm caterpillar *M. sexta* (Greenlee and Harrison 2005; Callier and Nijhout 2011, 2012; Sears et al. 2012). The general pattern is for the scaling relationship to flatten as animals grow within the instar, with essentially no relationship between metabolic rate and mass after larvae reach the critical weight for molting, even though larvae continue to accumulate mass during this time (Callier and Nijhout 2011). This pattern is indicative of metabolic rate becoming limited by O_2_ supply as larvae outgrow their tracheal system (Greenlee and Harrison 2005). Furthermore, while tracheal conductance is reset in each instar at molting to match or exceed O_2_ demand, mass-specific metabolic rates decrease significantly across instars (Greenlee and Harrison 2005; Callier and Nijhout 2011, 2012). This could be explained by a decrease in energy demand in all cells or by changes in the relative contribution of tissues with differing demands as insects develop. Callier and Nijhout (2012) found both a decrease in the proportion of highly metabolically active gut tissue in fifth-instar larvae and a decrease in Complex III (cytochrome c oxidase) activity across instars, supporting the idea that intrinsic metabolic demands change during ontogeny via both mechanisms. Similar patterns of inter-instar variation in scaling relationships and decreased cytochrome c oxidase activity in later instars have also been observed in the silkworm *Bombyx mori* (Blossman-Myer and Burggren 2010). One parameter that is missing from these studies is a measure of the RQ during development (for exception, see Blossman-Myer and Burggren 2010), which would indicate not only a change in tissue composition and energy demand, but also changes in what substrates are being used to fuel metabolism. Furthermore, we need more data documenting the dynamics of mitochondrial biogenesis and capacity as a function of larval development in insects (Blossman-Myer and Burggren 2010).

### Genetic effects on metabolic systems

In *D. melanogaster*, we have comprehensive transcriptome profiling of metabolic pathways across larval development, tissues, and environments (Chintapalli et al. 2007; Graveley et al. 2011) and metabolite profiling for larvae of different genotypes (e.g., Tennessen et al. 2011) and in different environments (e.g., Kostal et al. 2011); yet, we lack a detailed description of metabolic rate and its scaling with mass throughout ontogeny. Across *Drosophila* species, glycolytic enzyme activities are positively correlated with adults’ RMR (Berrigan and Hoang 1999), suggesting that datasets describing change in metabolic pathways and their metabolites may be critical in predicting metabolic rate during ontogeny and in response to the environment. What we do have in *D. melanogaster* is good evidence that there is significant genetic variation for mass-specific metabolic rate within species (Montooth et al. 2003; Hoekstra et al. 2013) and that mitochondrial–nuclear genotypes that specifically disrupt mitochondrial function, adversely affect metabolic rates and development of larvae (Hoekstra et al. 2013; Meiklejohn et al. 2013). With numerous genetic reference panels now available for which phenotypes can be measured for many natural genotypes (Greenberg et al. 2010; Jumbo-Lucioni et al. 2012; King et al. 2012; Mackay et al. 2012), *D. melanogaster* is well poised to further our understanding of genetic and genotype-by-environment effects on metabolic performance.

Recent genetic investigations in *D. melanogaster* highlight just how dynamic the balance between aerobic and anaerobic production of ATP may be across larval ontogeny. Tennessen et al. (2011) found that, during mid-embryogenesis, the *Drosophila* homolog of the estrogen-related receptor (dERR) regulates a key metabolic transition, promoting a metabolic program typically associated with cell proliferation, reminiscent of the Warburg effect associated with cancer cells (Warburg 1956; Vander Heiden et al. 2009; Cairns et al. 2011). This proliferative metabolic program upregulates carbohydrate metabolism, the pentose phosphate shunt, and anaerobic ATP production via lactate dehydrogenase and is critical for third instar larval survival (Tennessen et al. 2011). These pathways are then down-regulated in *Drosophila* and in *B. mori* during the instar preceding metamorphosis (Andres et al. 1993; White et al. 1999; Tennessen and Thummel 2011). Coupling this type of data with measures of mitochondrial capacity, VO_2_ and VCO_2_ across larval development will fill a critical gap in our understanding of how energy demands and supplies of O_2_ and substrates change during development.

An important remaining question is the extent to which changes in energy metabolism and metabolic rate across ontogeny reflect relative abundances of different types of tissues across larval development (Blossman-Myer and Burggren 2010; Callier and Nijhout 2012). Holometabolous insect larvae possess two distinct tissues, the proliferating imaginal tissues that will become the adult tissues, and the larval tissues, many of which are polyploid and rapidly accumulate mass and synthesize protein and other macromolecules (Kato et al. 1987; Edgar and Orr-Weaver 2001). These tissues likely have different demands for energy and usage of metabolic substrates that may drive ontogenetic and genetic variation in organismal metabolic rate.

### Plasticity of metabolic systems in response to the environment

**Fig. 4:**
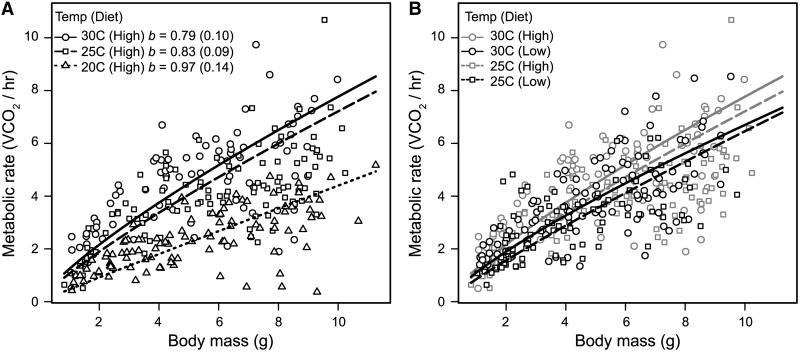
Temperature and diet interact to affect routine metabolic rate (RMR) in *Manduca sexta* caterpillars. Fifth-instar larvae were reared at one of three temperatures (30, 25, or 20°C) and one of two qualities of diet. VCO_2_ of larvae was measured at their rearing temperatures using a FoxBox Field Gas Analysis System (Sable Systems, Las Vegas NV). (**A**) Decreasing temperatures result in increasing values of the scaling exponent *b*, with *b* at 20°C not differing from 1. (**B**) While *b* did not differ between diets (High = standard diet, Low = 60% reduced diet), temperature and diet interacted to affect the scaling coefficient. At 30°C, but not at 25°C, larvae fed on a low-quality diet had significantly lower mass-specific RMR. See text for statistical results. Parameters were fitted using standardized major-axis regression after log10 transformation of both axes and were back-calculated to the power-law form for plotting. Values of *b* are provided with confidence intervals.

The thermodynamic effects of temperature on reaction rates, cell membranes, and metabolic and developmental rates in ectotherms are well studied (Krogh 1916; Sharpe and De Michele 1977; Behrens et al. 1983; Hazel 1995; Ruel and Ayres 1999; Clarke and Fraser 2004; Irlich et al. 2009; Waters and Harrison 2012). Temperature is also a major extrinsic factor that can affect metabolic scaling with mass [reviewed by Glazier (2005)]. Recent findings in *D. melanogaster* and *M. sexta* demonstrate that the temperature at which larvae develop impacts metabolic scaling. In *D. melanogaster*, larval development at 16°C results in a metabolic scaling exponent that is significantly greater than 1 (*b* = 1.27, 95% CI 1.068–1.508), while development at 25°C results in a metabolic scaling exponent that does not differ significantly from either 2/3 or 3/4 (*b* = 0.74, 95% CI: 0.64–0.85) (Table 2; Fig. 3B) (Hoekstra et al. 2013). Higher scaling exponents at lower development temperatures also have been observed in *M. sexta* larvae (Table 2; Fig. 4A) and other non-insectan invertebrates [reviewed by Glazier (2005)]. When reared at 20°C, *M. sexta* larvae have a scaling exponent that does not differ significantly from 1 (*b* = 0.97, 95% CI 0.83–1.11), with lower scaling exponents during development at higher temperatures (Table 2; Fig. 4A). *D. melanogaster* and *M. sexta* developed at lower temperatures, have considerably extended duration of development and increased body mass (Reynolds and Nottingham 1985; Zwaan et al. 1992; French et al. 1998; Ghosh et al. 2013). These new data (Table 2; Figs. 3B and 4A) indicate that intrinsic energy demands and supplies may increase with mass as a function of the temperature experienced during development, in contrast to the apparent decrease in demand for energy as larvae increase in mass throughout development (Callier and Nijhout 2012).

**Table 2:** Mass-scaling exponents for metabolic rates, either oxygen consumption (VO_2_) or carbon dioxide emission rate (VCO_2_), measured throughout ontogeny.

Parameter	Scaling exponent	Species	Developmental stages covered	References
VCO_2_	0.52	*Bombyx mori*	larval-prepupal	(Blossman-Myer and Burggren 2010)
VO_2_	0.82	*Bombyx mori*	larval-prepupal	(Blossman-Myer and Burggren 2010)
VCO_2_	0.98	*Manduca sexta*	larval instars 1–5	(Greenlee and Harrison 2005)
VCO_2_	0.95	*Manduca sexta*	larval instars 1–5	(Sears et al. 2012)
VO_2_	0.85	*Manduca sexta*	larval instars 3–5	(Callier and Nijhout 2012)
VCO_2_	0.97 (20°C)^a^	*Manduca sexta*	larval instar 5	Fig. 4A, this article
	0.83 (25°C)			
	0.79 (30°C)			
VCO_2_	1.27 (16°C)	*Drosophila melanogaster*	larval instar 3, pre-wandering	(Hoekstra et al. 2013)
	0.74 (25°C)			
VCO_2_	0.73	*Schistocerca americana*	larvae to adult	(Greenlee and Harrison 2004a)

^a^Developmental and measurement temperature is given when multiple temperatures were tested.

A striking result is that the ambient temperature during development alters how genotype affects both mass-specific metabolic rate and metabolic thermal plasticity (i.e., the *Q_10_* for metabolic rate) in *D. melanogaster* larvae (i.e., genotype–environment interactions) (Hoekstra et al. 2013). Larvae from two wild-type genetic strains (*OreR* and *Aut*) have significantly different mass-specific RMR when developed at 16°C, but not when developed at 25°C (Fig. 3C). However, at 25°C, a particular mitochondrial-nuclear genotype that decreases OXPHOS activity (Meiklejohn et al. 2013) has a significantly increased mass-specific RMR (Fig. 3C), consistent with inefficient production of ATP (Hoekstra et al. 2013). Furthermore, when reared at 25°C, this genotype has a normal *Q_10_* for metabolic rate, but when reared at 16°C this genotype has a significantly decreased *Q_10_* (Fig. 3D). These data indicate that metabolic thermal plasticity is not simply a function of thermodynamic effects, but it also depends on genotype and the interaction between genotype and environment.

Temperature also impacts the effect of diet on RMR in *M. sexta* larvae. Using flow-through respirometry to quantify VCO_2_ of fifth-instar larvae, reared on either a standard or a 60% reduced-calorie diet, revealed no impact of diet on the scaling exponent (test of common slopes, 25°C, *D* = 0.019, *P* = 0.89; 30°C, *D* = 0.37, *P* = 0.54; Fig. 4B). However, larvae had significantly decreased scaling coefficients (i.e., lower mass-specific RMR) on reduced calorie diets when reared at 30°C (test of common elevation, WALD = 7.82, *P* = 0.005), but not when reared at 25°C (WALD = 0.25, *P* = 0.62) (Fig. 4B). Statistical analyses were performed using standardized major-axis regression on log10 transformed data using the SMATR package (Warton et al. 2006) in R version 3.0 (R Core Team 2012). Dietary amino acids differentially impact the developmental progression for different types of tissue in *D. melanogaster* (Britton and Edgar 1998), and larvae fed on different diets may be differentially using different energy stores and pathways to generate ATP. Measures of tracheal volume, RQ, mitochondrial capacity, and energy storage and usage are needed to understand the proximate mechanisms underlying variation in metabolic performance of different genotypes across environments.

## The challenge: building predictive models of the development and plasticity of metabolic performance

Predicting the development and plasticity of metabolic performance in response to the environment will require measures of the O_2_ supply system, energy demand, and VO_2_ and VCO_2_ during development under ecologically relevant conditions. The community has made good progress on measuring different aspects of these systems during larval development in a few species of insects. However, to date, we have no complete dataset for any one taxon. An excellent question raised at the symposium was where to focus our efforts. *S. americana*, *D. melanogaster*, and *M. sexta* are the three species for which we have the most complete datasets. Ideally, we would have phylogenetically independent data showing how tracheal supply and metabolic demands vary throughout development. More immediately, having complete datasets for the three species mentioned above, from at least one other hemimetabolous insect (e.g., cockroaches) and from an aquatic insect would be significant progress.

**Fig. 5:**
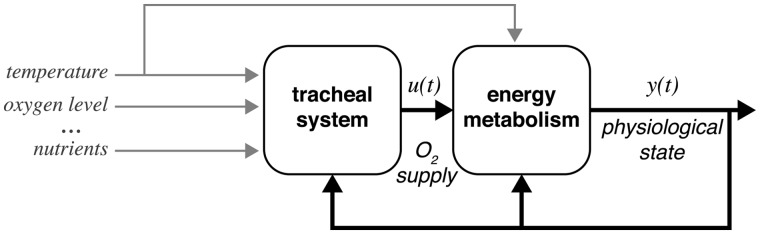
Possible experimental topology for a closed-loop model of metabolic performance. Blocks represent subsystems, which are typically modeled by a system of differential equations (or transfer functions) that capture the dynamics of the underlying biological components, connected by signals, represented by arrows. Gray arrows represent potential environmental signals that can be experimentally manipulated to affect the outputs of the system either on short (fluctuations in the environment) or long (developmental) time-scales. Environmental signals may also impact the two subsystems independently, as indicated here for temperature. The topology is based on model topologies describing neural control of locomotion, with thicker lines representing potentially higher dimension signals (Roth et al. 2014).

We have equations to relate tracheal volumes to O_2_ delivery, and several contributions to this symposium have highlighted methods to model organismal and cellular performance as a function of metabolic pathways (Ciaccio et al. 2014; Nijhout and Reed 2014). A challenge will be to employ a quantitative framework that can interface the systems of supply and demand to understand how metabolic performance is homeostatic to maintain a physiological state throughout development in response to transient environmental perturbations but can also account for plastic responses that remodel tracheal systems or metabolic pathways in response to persistent change in the environment. Dynamic energy budget (DEB) theory provides a strong quantitative framework, based on models of how organisms acquire and use energy, for linking processes at different scales of biological organization and predicting performance of individuals in a given environment (Nisbet et al. 2000; Sousa et al. 2010). Control theory has been successfully used as a computational framework that uses closed-loop control to predict systems’ behavior in a few key areas of biology, such as neurophysiology, biomechanics, and locomotion (Cowan et al. 2014; Roth et al. 2014). An appeal of control theory is that it incorporates the idea that system-level behavior is governed by feedback control, without which many systems would be unstable. Thus, this framework should apply to many physiological systems (e.g., the homeostatic regulation of ATP). Furthermore, Roth et al. (2014) argued that this computational framework can be used to generate hypotheses and guide experimental approaches. The control theory framework is a top–down approach to modeling in which subsystems are represented as input–output blocks, each of which is typically modeled by a system of differential equations (or transfer functions) that capture the dynamics of the underlying biological components, connected by signals represented by arrows (Fig. 5). This has an appeal for complex systems like metabolic performance, for which there may be many closed loops that govern the system’s behavior and for which we do not have measures for all the components required to build a model from the bottom up. The hope is that this top–down computational framework will incorporate new data across scales of biological organization (e.g., molecules, cells, structures, and organisms) to improve upon present feedback models and generate new hypotheses to investigate the underlying mechanisms that govern variation in metabolic rate and plasticity in response to the environment.

The application of control theory to this system of change over developmental time is complex, and nonlinear dynamics may become more important over developmental timescales, in contrast to the relatively short timescales of biomechanical and locomotor responses to rapidly changing environmental cues (e.g., Dyhr et al. 2013; Madhav et al. 2013). In addition, to describe change in systems over developmental time, these models will need to integrate information across scales of function. How to incorporate developmental trajectories for both the tracheal supply system and the metabolic output is challenging, because we do not fully understand the extent to which the demand for energy will feed back to shape the oxygen supply system and how the oxygen supply will result in remodeling of the pathways underlying metabolism. Furthermore, incorporating genetic and gene-by-environment variation into organismal models will be critical for making accurate models. Incorporating genetic variation of humans into models of metabolic networks has revealed the mechanisms by which these systems achieve stability (Nijhout and Reed 2014); the data we review above indicates that incorporating genetic variation will be critical for predicting metabolic stability and plasticity. Incorporating genetic variation into quantitative models of organismal function allows mapping of genotypes onto phenotypes, which is critical for understanding how organismal function evolves.

Finally, we need a better understanding of the sensors, controllers, and regulators—important components for modeling how respiratory structures and metabolic systems achieve stability and respond to the environment. Animals could be sensing ADP/ATP levels at the cellular level, sensing tissue oxygenation, or sensing the environment directly (e.g., oxygen level, temperature, or nutrient resources). Controllers could include the nervous system and the endocrine axis. Hormonal control of insect development and metamorphosis is well characterized (Nijhout 1994; Riddiford 1994; Flatt et al. 2005), and these hormones can also impact mitochondrial distributions, and presumably aerobic metabolic capacity, in tissues during development (Bradley 1984). Candidate genes regulating respiratory and metabolic systems include HIF signaling (Centanin et al. 2008, 2010) and the ERR nuclear hormone receptor, which has recently been implicated as a master regulator of mitochondrial biogenesis and function (Eichner and Giguere 2011) and of carbohydrate metabolism in *D. melanogaster* larvae (Tennessen et al. 2011; Tennessen and Thummel 2011). In *D. melanogaster,* dERR is also required for HIF and HIF-independent hypoxic responses (Li et al. 2013).

## Extensions to higher scales of biological organization

In closing, we note that while this discussion has focused on understanding variation in metabolic rate and mass-scaling within and among individuals, exciting progress has been made on the other end of the spectrum of biological organization. Population and community metabolic rates have been measured in at least three social insect species (Southwick et al. 1990; Shik 2010; Waters et al. 2010), all of which exhibit hypometric scaling of metabolic rate with mass, despite the fact that they are made up of physiologically independent individuals and should thus scale linearly with mass (Waters and Harrison 2012). Waters and Harrison (2012) argue that investigation of metabolic rates of an entire colony may be a powerful approach to understanding mechanisms underlying metabolic-scaling relationships because colonies can be empirically dissected into component parts. This provides an interesting complement to the mechanistic dissection of systems of supply and demand within and among individuals that we propose here for understanding stability and plasticity in metabolic performance.

## Funding

The Symposium was supported by NSF IOS-1243801 (to D.K. Padilla). This work was also supported by NSF IOS-0953297 and NSF MRI-1229417 (to K.J.G.) and NSF IOS-1149178 (to K.L.M.).
